# Genetic Factors Underlying Single Fiber Quality in A-Genome Donor Asian Cotton (*Gossypium arboreum*)

**DOI:** 10.3389/fgene.2021.758665

**Published:** 2021-12-07

**Authors:** Muhammad Shahid Iqbal, Shurong Tang, Zareen Sarfraz, Muhammad Sajid Iqbal, Hongge Li, Shoupu He, Yinhua Jia, Gaofei Sun, Zhaoe Pan, Geng Xiaoli, Abid Mahmood, Saghir Ahmad, Mian Faisal Nazir, Baojun Chen, Liru Wang, Baoyin Pang, Shoujun Wei, Xiongming Du

**Affiliations:** ^1^ State Key Laboratory of Cotton Biology/Institute of Cotton Research, Chinese Academy of Agricultural Sciences (ICR, CAAS), Anyang, China; ^2^ Ayub Agricultural Research Institute Faisalabad, Cotton Research Institute, Multan, Pakistan; ^3^ Khwaja Fareed University of Engineering and Information Technology, Rahim Yar Khan, Pakistan; ^4^ Anyang Institute of Technology, Anyang, China

**Keywords:** GWAS, AFIS, fiber quality, SNPs, Asian cotton, multi-environment

## Abstract

The study of A-genome Asian cotton as a potential fiber donor in Gossypium species may offer an enhanced understanding of complex genetics and novel players related to fiber quality traits. Assessment of individual fibers providing classified fiber quality information to the textile industry is Advanced Fiber Information System (AFIS) in the recent technological era. Keeping the scenario, a diverse collection of 215 Asiatic cotton accessions were evaluated across three agro-ecological zones of China. Genome-Wide Association Studies (GWAS) was performed to detect association signals related to 17 AFIS fiber quality traits grouped into four categories viz: NEPs, fiber length, maturity, and fineness. Significant correlations were found within as well as among different categories of various traits related to fiber quality. Fiber fineness has shown a strong correlation to all other categories, whereas these categories are shown interrelationships *via* fiber-fineness. A total of 7,429 SNPs were found in association with 17 investigated traits, of which 177 were selected as lead SNPs. In the vicinity of these lead SNPs, 56 differentially expressed genes in various tissues/development stages were identified as candidate genes. This compendium connecting trait-SNP-genes may allow further prioritization of genes in GWAS loci to enable mechanistic studies. These identified quantitative trait nucleotides (QTNs) may prove helpful in fiber quality improvement in Asian cotton through marker-assisted breeding as well as in reviving eroded genetic factors of *G*. *hirsutum via* introgression breeding.

## Introduction

Cotton has a prime position in the global natural textile fiber industry, making it a significant agricultural commodity. It remains an essential source of livelihood for a large percentage of the farming community. Cotton production has long been a crucial part of diverse farming systems, particularly those involving vegetables and cereals, helping farmers to maintain their incomes. The competition among major cotton-producing regions and the use of synthetic fibers are continuously increasing. It necessitates continual product quality improvements, measuring physical traits and germplasm resources (Meredith Jr, 2005). Almost 90% of the significance of the cotton crop relies on its lint fiber. Generally, each fiber is an elongation (seed hair) originating from a cotton ovule, from protodermal cells in the ovule’s seed coat integument (outer) layer. Single seed fibers are assumed to exhibit continua of shape, physical maturity, cell wall thickness, and length (Bradow et al., 1996). These physical traits determine the quality of the raw material, which underlie the quality of the finished product. However, determining cotton fiber quality is complex. As cotton fiber quality is a critical issue in cotton research, an accurate and precise measurement system is required for the various fiber quality traits (Berkley, 1948).

For the past two decades, industry and plant breeders have been utilizing High Volume Instrument (HVI) as the primary and sole source of measurement for selection and fiber quality improvement. However, the HVI system cannot assess many key fiber quality traits. Alternative systems for fiber quality evaluation, such as the Advanced Fiber Information System (AFIS), have been introduced. AFIS can obtain highly advanced and more accurate information about single fiber quality. Many studies have indicated that AFIS is an effective tool for predicting yarn quality along with spinning performance (Hequet et al., 2006). AFIS is the instrument of choice in the cotton industry, including cotton breeders, based on its ability to estimate mean fiber values and distributions. It accurately measures maturity and fineness through cross-sectional image analysis. Hence, AFIS is a powerful tool for the industry if appropriately linked with image analysis data (Thibodeaux et al., 2007).

*Gossypium hirsutum* (upland cotton), the most widely cultivated cotton species, is considered a natural allotetraploid cotton species with an AADD genome. It is thought to have resulted from natural interspecific hybridization involving the diploid species *Gossypium arboreum* (genome A2) and *Gossypium raimondii* (genome D5) (Paterson et al., 2012). It is challenging to explore the two co-resident genomes in *G. arboreum* that have unverified origins, and these tetraploid species are challenging to study. However, The A-genome donor of *G. hirsutum*, i.e., *G*. *arboreum*, harbors many putative genetic factors underlying fiber quality traits and stress resistance, and an in-depth study of *G*. *arboreum* might provide insights that could help to improve the *G*. *hirsutum* yield and fiber traits. Many cotton breeders and cotton geneticists are currently trying hard to understand the two-donor diploid genomes thoroughly. To understand mechanisms underlying fiber quality traits in the diploid species, it is essential to identify the genes controlling these traits. It may aid the introgression of genetic factors from diploid to tetraploid cotton.

In previous decades, *G*. *arboreum* cultivars rather than tetraploid cultivars were commercially grown in north-eastern Africa, the Middle East, and Asia (Guo et al., 2006). Being a diploid species, *G*. *arboreum* is highly adaptable to extreme environmental conditions (Maqbool et al., 2010) and can be cultivated using practical management approaches and fewer inputs (Iqbal et al., 2015). Its valuable traits include strengthened fiber, high seed index, and high oil content (Mehetre et al., 2003). Additionally, resistances against biotic stresses viz; reniform nematode (Erpelding and Stetina, 2013), tobacco budworm (Hedin et al., 1992), Cotton leaf curl virus (CLCuV) (Nazeer et al., 2014), and thrips (Stanton et al., 1992) can possibly be introgressed to *G*. *hirsutum*. However, specialized breeding techniques would be required to overcome the barriers during the hybridization process (Sacks and Robinson, 2009).

Information on the variability among *G*. *arboreum* genotypes and the complex interactions among valuable traits may allow improved cotton breeding programs to be developed. Predicting genotype variability can be accomplished *via* phenotypic assessments and characterization (Tahir and Noor, 2011). To develop a breeding program, the degree of potential within the genotypes in question and the extent of the associations among the target traits should be evaluated (Batool et al., 2010; Khan et al., 2010). There are four industrially important categories of fiber quality traits, namely, fineness, maturity, NEPS, and complete length. During the 20th and 21st centuries, many classical quantitative genetic studies calculated the variance and heritability of yield and fiber traits and their interactions with environmental factors, leading to yield and quality advances. However, the yield and quality have stagnated over the last decade, which may be due to the phenotypic selection pressure placed on commercial *G*. *hirsutum* cultivars*.* It may have ultimately reduced the genetic diversity in the primary cotton gene pool. It may explain the increase in the vulnerability of *G*. *hirsutum* cultivars to biotic and abiotic stresses (Maqbool et al., 2010). Classical cotton breeding efforts involving interspecific hybridizations for stable genetic transformation of novel allelic variation have encountered challenges. However, many related genomic tools and biological procedures have been developed. The significant advancements include polymorphic genetic markers, linkage maps, and divergent mapping populations.

Keeping the scenario, this study’s primary goals include identifying genes and residing regulatory sequences controlling cellulose biosynthesis and cell development of fiber. For sequencing, assembling, and annotation of *G*. *arboreum* genome, contemporary genomic resources will be established. A genome-wide association study (GWAS) was conducted to correlate phenotype data of fiber quality traits taken from AFIS with genotypic data generated from Next-Generation Sequencing (NGS) technology. Using different algorithms while performing GWAS, key SNPs and fiber quality associated genes were identified and selected. A thorough analysis of functional annotation *via* bioinformatics tools was utilized to confirm the linkage between genes and traits in diploid Asian cotton genotypes.

## Materials and Methods

### Plant Materials

Asian cotton (*G*. *arboreum*) collection having 215 accessions (Supplementary Table S1) were obtained from the Chinese National Germplasm Mid-term Genebank (Institute of Cotton Research, Anyang, China). They were grown in three diverse ecological regions of China in 2014, and their fiber quality was evaluated. The three regions were as follows: two major conventional cotton-growing regions, i.e., Anyang, Henan (Yellow River Region), and Akesu, Xinjiang (Northwest Region), and a non-conventional potential cotton region, i.e., Sanya, Hainan (an island in South China). The experiment involved a triplicate randomized complete block design. Row spacing was 70 cm, and plant spacing was 30 cm in all locations. Planting was conducted during the regular cotton growing season (April) in Anyang and Akesu. However, in Sanya, planting was conducted during an extended cotton growing season (October–March). The agronomic and cultural practices were uniform across all locations to avoid biasness. Five guarded plants were randomly tagged for genotyping, along with phenotyping. At physical maturity, seed cotton was manually picked from each tagged plant separately. Muslin cloth bags were used for each sample to avoid mixing or any type of adulteration.

An AFIS PRO 2 (Zellweger-USTER) was used to assess the cotton fiber traits, including their distributions, which were presented using histograms of the distribution for the measured parameters (Shofner et al., 1990), including NEPS, fiber length, maturity as well as fiber fineness. The 17 traits assessed were as follows: Total nep count (TNN), Total nep mean Size (TNS), Fiber nep count (FNN), Seeds oat nep Count (SCN), Seed coat nep size (SCS), Mean length Weight (LW), Length Weight Variation (LWCV), Short Fiber Content Weight (SFCW), Upper Quartile Length Weight (UQLW), Fiber Length Variation (LNCV), Mean Length Number (Ln), Short Fiber Content (SFCn), The 5% Length Number (Ln5) and Short Fiber Content Number (SFCN), Maturity Ratio (MR), Immature Fiber Content (IMM), and Fiber Fineness (Mtex) detailed information about these traits is given in Supplementary Table S2.

The AFIS PRO 2 mechanically separates individual cotton fibers presented to an electro-optical sensor using high-velocity airflow. The AFIS Length & Maturity module optically determines the value for maturity ratio (MR) using Lord’s equation while Immature Fiber Content (IMM) through the method described by Frydrych and Thibodeaux (2010). The AFIS PRO2 (which is the latest version) can complete one test in 2.5–3 min (Frydrych and Thibodeaux, 2010). After drying and cleaning, ginning was conducted to obtain lint samples for fiber quality analysis. The single fiber quality-related traits for NEPS, length, fineness, and maturity (Supplementary Table S2), were assessed three times for each sample to avoid error and then averaged for further statistical analysis.

### Preparation for GWAS

The genotype data, which was above an average 6-fold sequencing depth, were derived from a previously published article by our team (Du et al., 2018), and the sequence of the same 215 (GA0001 to GA0215) accessions was considered for phenotyping in this experiment was picked out and used in this study. The genotype data were filtered before GWAS, SNPs with MAF <1% and SNPs that exhibited deviations from Hardy–Weinberg equilibrium (HWE) were removed, leaving 1,425,002 SNPs for GWAS. HWE theorem states that both the allele and genotype frequencies in a population remain constant, so testing for HWE is a standard quality control procedure in population genetic studies. Due to the large number of SNPs, subsets were analyzed separately; for this purpose, we employed PLINK v1.07, an open-source GWAS toolset (Purcell et al., 2007), using an R plugin. In a GWAS, while we hope for some true associations, most of the SNPs are not associated with the trait in question, so almost all *p*-values should come from a uniform distribution.

SNP annotation information was generated *via* utilizing ANNOVAR software (Wang et al., 2010) based on the *G*. *arboreum* reference genome. The genomic regions were categorized into different groups using genome annotation such as: downstream or upstream regions, the annotated SNP lying within 1 kb region either downside of transcription stop site/upside of transcription start site OR upside and downside of transcription sites simultaneously; intronic (non-coding) region; exonic (coding) region; splicing sites, lying within 2bp of splicing junction; and intergenic regions. Further, the SNPs harbored by the exonic regions were categorized as synonymous (didn’t cause any change in amino acids); non-synonymous (caused changes of amino acids); as well as stop-gain and stop-loss type of mutations also grouped in this category.

### Statistical Analyses

The data collected were subjected to multivariate analysis using hierarchical clustering analysis and principal component analysis (PCA). After confirming sufficient variation among the traits, GWAS was conducted, making the traits suitable for further genetic analyses. Multivariate correlation analysis was performed to assess the relationships among the 17 traits. The analyses were performed using JMP Pro 14.0 software (SAS Institute Inc.).

### Phylogenetic and Population Structure Analyses

To understand the phylogenetic relationships among the accessions, phylogenetic analysis was performed using the SNPhylo pipeline (Li et al., 2014). The SNPs were pruned to reduce SNP redundancy due to linkage disequilibrium (LD; SNPs in a specific LD block provide redundant lineage information). SNPhylo uses only one informative SNP in each LD block, so the phylogenetic analysis was based on high-quality SNPs. A subset of 707,968 high-quality SNPs [>2000, minor allele frequency (MAF) >5%], with missing data rate <20%, was utilized. A neighbor-joining tree was constructed using PHYLIP v3.696 (Felsenstein, 1993), with 100 bootstrap replicates.

To infer the population structure, ADMIXTURE software was used (Falush et al., 2003). This software uses a mode-based clustering method that considers different numbers of clusters (*K*). A set of 431,985 SNPs, excluding missing genotypes, was included in the analysis. Additionally, SMARTPCA in EIGENSOFT software (Patterson et al., 2006) was used to conduct PCA on a set of 4,329,838 SNPs.

### GWAS

A set of 1,425,002 high-quality SNPs (MAF >5%, <20% missing rate) from 215 diverse *G*. *arboreum* accessions were used for the GWAS. The GWAS was conducted using EMMAX (Efficient Mixed-Model Association eXpedited) software (Kang et al., 2010), which can handle large datasets for GWAS. The 17 AFIS-related fiber quality traits in three ecological locations/environments (Anyang, Henan; Sanya, Hainan; and Akesu, Xinjiang) were considered in this analysis. Population stratification and hidden relatedness were modeled with a kinship (*K*) matrix using the EMMAX-kin-intel EMMAX package. Bonferroni correction (dividing the desired *p*-value by the number of comparisons (Yang et al., 2005; Pearson and Manolio, 2008) was used to avoid a high false-positive rate. Hence, the adjusted *p*-value threshold for the *G. arboreum* accessions was *p* < 4.9 × 10^–5^. Manhattan plots and quantile-quantile (Q–Q) plots were constructed using the CMplot R package to visualize the results. Lead SNPs were identified from the Manhattan plots by selecting the SNPs from each peak having the higher −log*P* = 6.15 using formula P < P = 1/N (where N is the total number of SNPs used for GWAS.

### LD Analysis

Linkage Disequilibrium (LD) decay was visualized by plotting *r* ^2^ against the physical distance (kb) between the SNPs using PopLDdecay software (Zhang et al., 2019). The GWAS results related to four chromosomes (Chr02, Chr03, Chr05, and Chr06), and the four trait categories are depicted in detail (Figures 5–8). The LD (D′) decay distance between the paired genes and SNPs ranges 115.5 kb (57 kb on either side of each key SNP). The GWAS and LD combined plot of each selected area were plotted using LDBlockShow software (Dong et al., 2021). All genomic positions provided were based on the *G*. *arboreum* reference genome (Li et al., 2014) v1.1. Differential expression data regarding the genes near lead SNPs were obtained from our Institute database (http://grand.cricaas.com.cn/) and the Cotton Functional Genomics Database (www.cottonfgd.org). The upregulated genes (>1) specially in ovule and fiber were considered candidate genes and plotted in a heatmap.

## Results

### Phenotypic Characteristics

The following 17 phenotypic single fiber quality traits of 215 diploid *G*. *arboreum* accessions were assessed (Supplementary Table S2): NEPs (TNN, TNS, FNN, FNS, SCN, SCS), maturity (MR and IMM), fineness (MTex), and length (LW, LWC, Ln, Ln5, LCV, SFCW, SFCn, and UQLW). A considerable range of variation and normal distribution with insignificant skewness and kurtosis has been illustrated regarding investigated fiber quality traits under three different environments (Supplementary Figure S1, Supplementary Table S3). Normal distribution values in the diagonals and range of variation *via* boxplots on the extreme right of the scatterplot matrix has been shown in Supplementary Figure S1 provided sufficient grounds for further processing of data for GWAS (Supplementary Table S3). The highest standard deviation (Std Dev) has been displayed by NEPs related trait followed by length related traits and lowest Std Dev was exhibited by maturity related trait. Similar trend was followed by traits across all locations and further detailed description has been provided in Supplementary Table S3.

**FIGURE 1:**
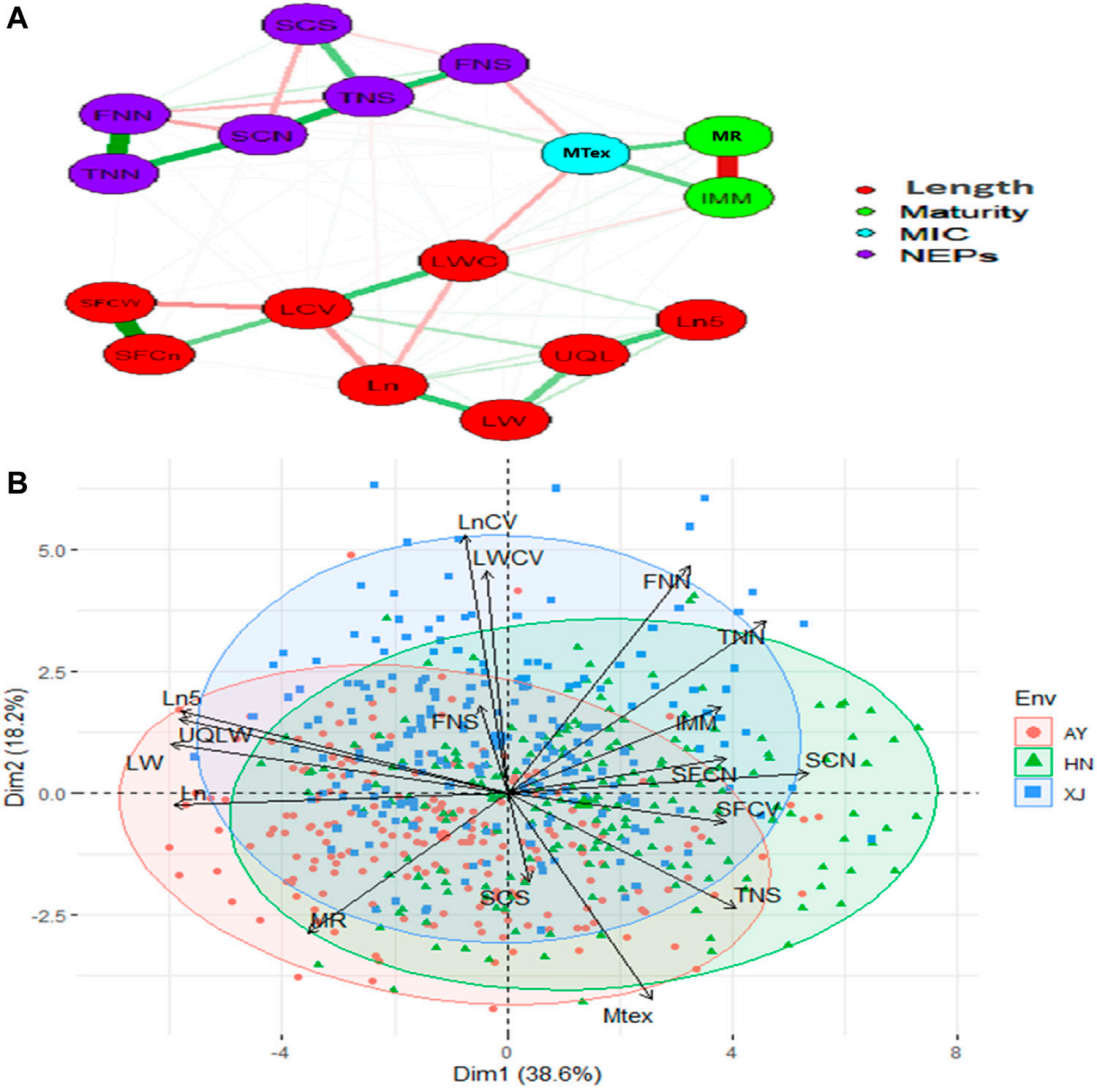
Relationship network and PCA biplot of AFIS Relate Fiber Quality Traits **(A)** Relationship network between different single fiber quality traits of *G*. *arboreum* accessions. Red and green color connection lines between each trait illustrate negative and positive relationships between them, respectively. All the investigated traits are categorized into four major groups of Length (Red), Maturity (Green), MIC (Blue), and NEPs (Purple) **(B)** PCA-based biplot on the phenotypic variation of single fiber quality traits projected in the PCA1 and PCA2 planes. Legends on the top right: Three different colors represent different environments in which G. arboreum accessions were evaluated; Black to red color gradient represents the contribution of each trait in variation.

The strength and direction of correlations among the various traits are shown in the scatterplot matrix in Supplementary Figure S1. The upper values in black represent the overall correlations. The lower values in different colors represent the correlations in each location. Almost all the correlations were statistically significant. The significant or highly significant positive correlations were determined among SFCn, SFCW, TNN, FNN, SCN, TNS, Ln, LN5, LW, UQLW. However, highly significant negative correlations were among MAT, IMM, SFCW, SFCn, SCN, Ln, Ln5, LW, LCV, LWC and UQLW (Supplementary Figure S1). PCA was also used to investigate the relationships among the phenotypic traits and the factors underlying trait variation. The first two principal components (PCs) explained 56.8% of the total variation of the traits. PC1 explained 38.6% of the total variation, and loading on this PC was highest for FNN, LWC, LCV, and Mtex. Both FNS and SCS exhibited maximum loadings on PC4 (Figure 1).

### Population Structure, PCA, Phylogenetic, and LD Analyses

**FIGURE 2:**
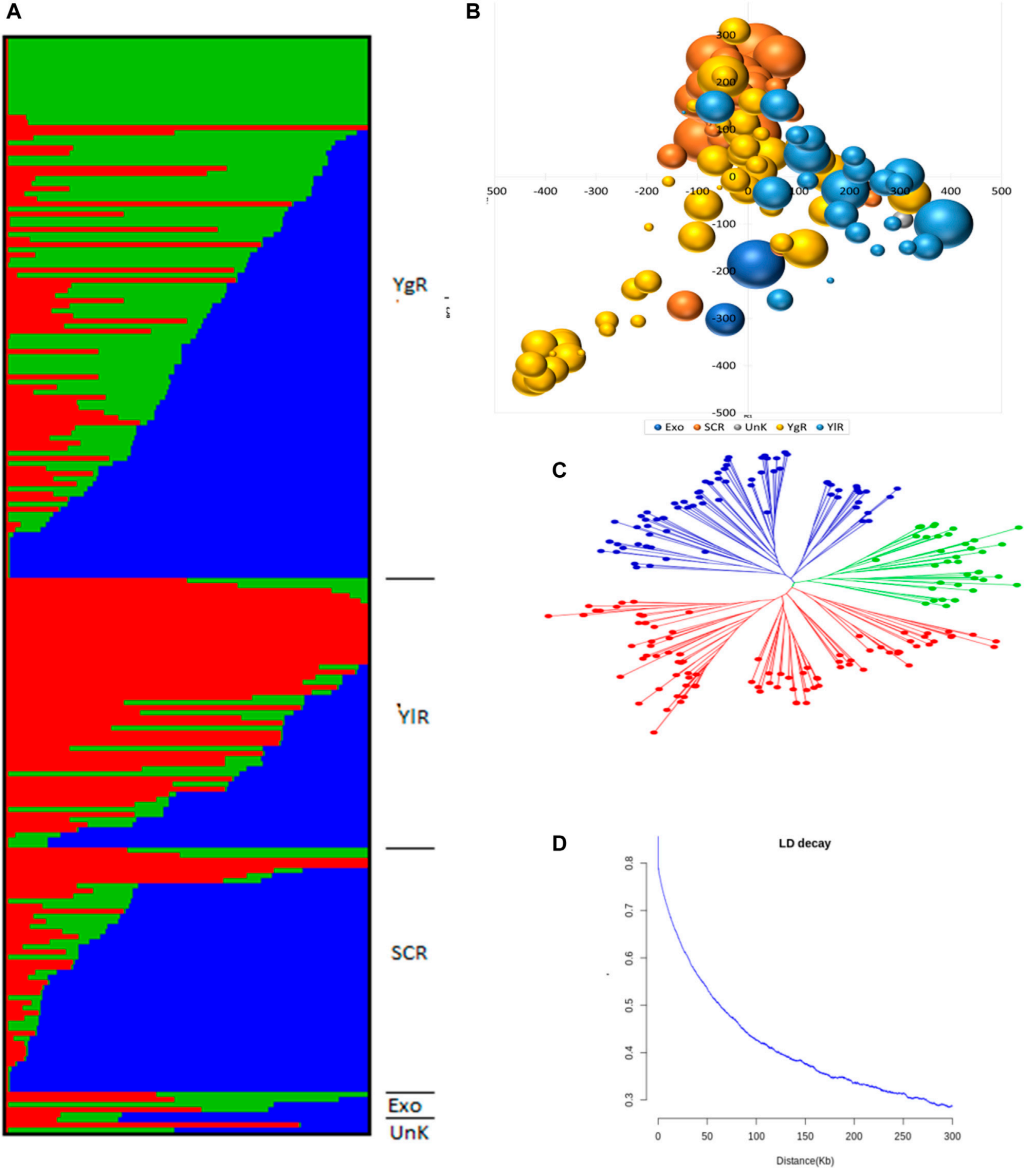
Population Genetic Study **(A)** Population structure of 215 *G*. *arboreum* cotton accessions in the association panel based on modeled ADMIXTURE analysis at K = 3. The component structural groups of Asiatic cotton are divided into cotton growing zones i.e., YgR (Yangtze River Region), YlR (Yellow River Region), SCR (South China Region), Exo (Exotic) and UnK (Unknown) **(B)** Principal component analysis (PCA) plot of PC1 and PC2 based on 1430005 genomewide SNPs scored on 215 *G. arboreum* accessions **(C)** Phylogenetic tree constructed using whole-genome SNP data, distributing genotypes into three clades as per original classification **(D)** Decay of linkage disequilibrium (LD) expressed as a function of physical distance (kb) and *r* ^2^.

A set of 215 *G. arboreum* lines were evaluated across two major conventional cotton-growing regions (Anyang and Akesu) and one non-conventional (Sanya) cotton-growing region in China for association studies. Population structure, PCA, and phylogenetic analyses (Figures 2A–C). The phylogenic tree indicated the division of accessions into YZR, SC, and YER groups, revealing substantial geographical distributions (Figure 2C). PCA confirmed this clustering. Also, high nucleotide diversity was observed, as compared to and within clusters. LD analysis is a helpful tool to locate causal loci in GWAS, and we found that the LD decay distance in the *G*. *arboreum* accessions was ∼115.5 kb (indicating the physical distance between SNPs) (Figure 2D). These findings indicate a significant population structure in these accessions.

### GWAS of AFIS Fiber Quality Traits

Halting the stagnation of fiber quality has been the primary objective of cotton breeding programs in the last few decades. GWAS was performed on the 215 *G*. *arboreum* accessions evaluated across three locations to identify genetic factors linked to single fiber quality traits while considering population structure and phylogenetic relatedness (Yu et al., 2006).

A set of 1,425,002 high-quality SNPs with MAF >5% were utilized for GWAS (Supplementary Table S4). The EMMAX software detected 10,434 association signals with a threshold probability value *p* < 4.9 × 10^–5^ (Supplementary Table S5). For the first time, such associations were identified for single fiber quality traits evaluated across multiple environments (Supplementary Table S6). The distribution of 7,429 identified and annotated significantly associated SNPs is presented in Table 1 which were categorized into different regions or groups. Among these SNPs, 4,328 were grouped into the intergenic (non-coding) regions and 3,101 annotated SNPs were grouped in the genic (coding) regions with a division detail as: 766 significantly associated SNPs of upstream region; 760 downstream region SNPs; 509 upstream/downstream regions SNPs and 339 exonic SNPs with 164 non-synonymous, 161 synonymous, 1 splicing, 7 stop codon loss, 6 stop codon gain SNPs which triggered amino acid changes and premature stopping or elongated transcripts production (Table 1).

**TABLE 1:** Counts of significantly associated SNP for 215 *G*. *arboreum* genotypes across three geographical locations.

Genomic region	SNP count	Percentage
Intergenic	4328	58.26
Genic	3101	41.74
Intronic	727	9.79
Upstream	766	10.31
Downstream	760	10.23
Upstream; downstream	509	6.85
Exonic (non-synonymous)	164	2.21
Exonic (synonymous)	161	2.17
Exonic (stop codon loss)	7	0.09
Exonic (stop codon gain)	6	0.08
Splicing	1	0.01
Grand total	7,429	100.00

**FIGURE 3:**
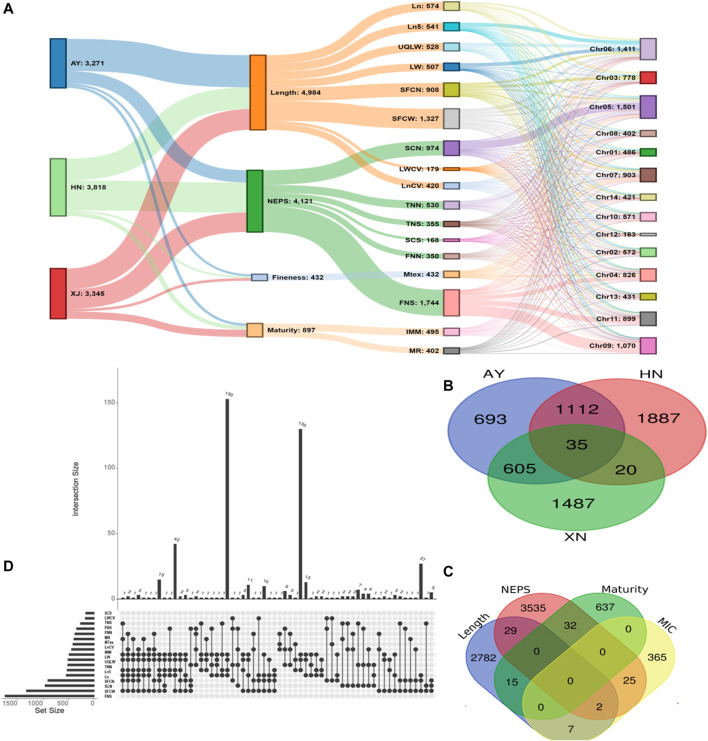
GWAS Association Summary **(A)** Sankey diagram illustrating the number of significantly (*p* < 0.00001) associated SNPs among evaluated environments (AY: Anyang, HN: Sanya, Hainan, XJ: Xinjiang) and single fiber quality traits along with their distributions 13 chromosomes of Asiatic cotton **(B)** Venn diagram displaying the number of significantly (*p* < 0.00001) associated SNPs shared among three evaluated environments **(C)** Venn diagram depicting number of significantly (*p* < 0.00001) associated SNPs shared among four major categories of studied single fiber quality traits of *G*. *arboreum* accessions **(D)** Details of significantly associated SNPs showing pleiotropism among various investigated single fiber quality traits. All SNPs with one or more associated phenotypes are shown here.

There were 3271, 3816, and 3345 SNPs significantly associated with single fiber quality traits AY, HN and XJ, respectively (Figure 3A, Supplementary Table S7). There were 35 SNPs common to all three locations, 1112 common in AY and HN, 605 common to AY and XJ, and 20 commons to XJ and HN (Figure 3B). 4984 SNPs were associated with length traits, 4121 with nep traits, 432 with fineness traits, 897 with maturity traits (Figure 3A). Two SNPs were associated with length, fineness, and nep traits, 29 with length and nep traits, 15 with length and maturity traits, 7 with length and fineness traits, 32 with nep and maturity traits, and 25 with fineness and maturity traits (Figure 3C).

**FIGURE 4:**
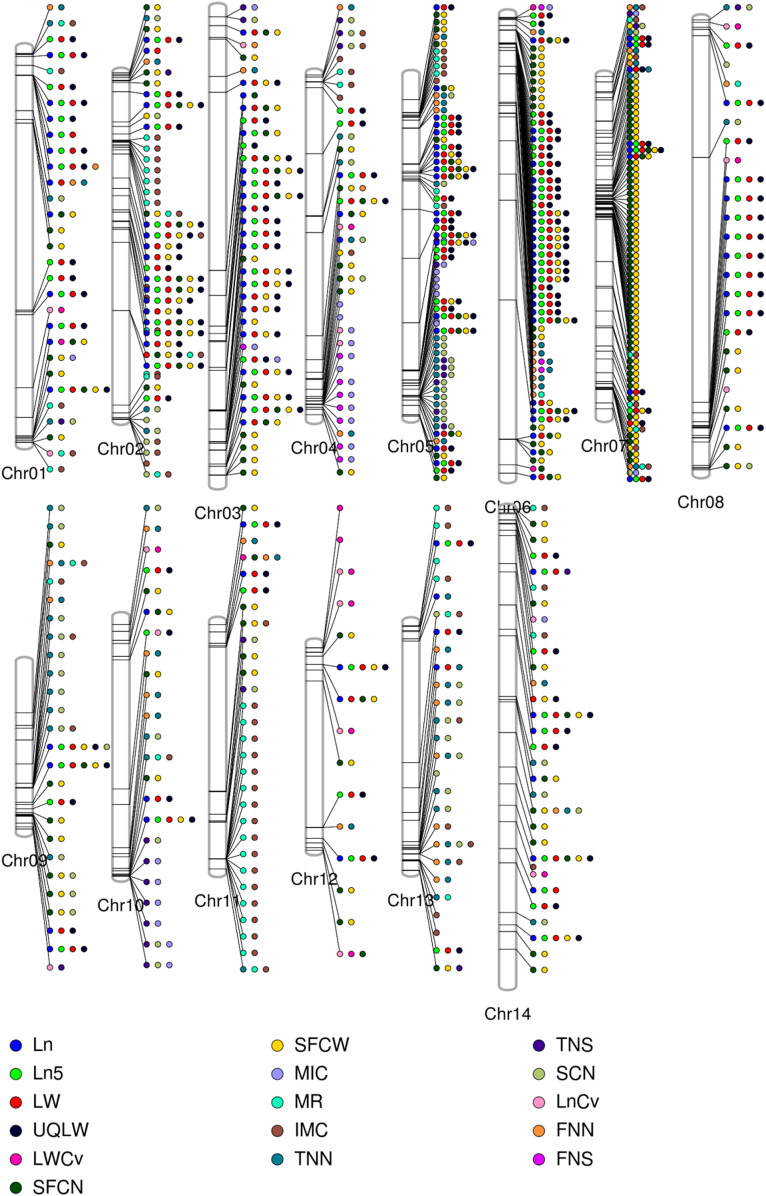
Phenogram is displaying peak associations signals from 7429 significantly associated SNPs for single fiber quality traits across different chromosomes of Asiatic Cotton. Legends at the bottom are for distinguishing different studied traits.

A total of 7,429 of the significant trait-associated SNPs distributed on the 13 chromosomes of diploid *G*. *arboreum* accessions were located in quantitative trait nucleotide (QTN)-rich regions (Supplementary Figure S3). The detailed chromosomal distribution is presented in Figure 3A. The maximum number of associations were on chromosome 5, while the minimum on chromosome 12 (Figure 3A). A similar trend of peak associations hits has also been observed on different chromosomes and represented in Figure 4.

A trend of pleiotropy was discovered for significantly associated SNPs. Out of 7,429 significant SNPs, 1,852 SNPs displayed pleiotropy for single fiber quality traits (Figure 3D). A sum of 228 association signals were observed based on multiple corrections (1/1430002 = 6.99E-07 or ∼0.000001 value) for 177 Lead SNPs (−log*P* = 6.15) for 14 single fiber quality traits across three environments with 155 association signals detected from AY, 47 from HN and 26 from XJ. Thorough examination revealed that chromosomal distribution of lead SNPs is uneven across the entire genome. Chromosome 5 had a maximum number of 65 lead SNPs while Chromosome 12 had a minimum of 1 lead SNP (Supplementary Tables S8, S9).

### Candidate Gene Prediction and Annotation

A total of 113 genes were discovered in the 115.5 kb flanking window of lead SNPs. Of these genes, 25 and 1 were identified in maximum and minimum quantities on Chromosome 7 and Chromosome 12, respectively (Supplementary Table S10). These genes were further scrutinized based on their differential gene expression data from our Institute database (http://grand.cricaas.com.cn/) and the Cotton Functional Genomics Database (http://www.cottonfgd.org/) (Supplementary Figure S3; Supplementary Tables S10A,B). As a result, 56 candidate genes were identified *via* validation from gene expression data of various cotton tissues, organs, or growth and developmental stages (Supplementary Table S12). Of these 56 genes, 28 were associated with length, 25 with NEPs, 1 with maturity and 2 with fineness (Supplementary Table S11). Further, Gene Ontology (GO) annotation (cellular components, biological processes as well as molecular functions) of putative genes was performed (Supplementary Table S12). The GWAS summaries of lead SNPs related to NEPs, maturity, fineness, and length comprised of Manhattan plots with a significance threshold horizontal line drawn at [−log (*p*) > 6], quantile-quantile plots, GWAS-LD blocks for the depiction of haplotype region surrounding the peak associated significantly with respective trait coupled with regional plot revealing particular key SNP and its nearby gene, a boxplot for the demonstration of differential among trait related favorable haplotypes as well as expression plots of prominently selected annotated genes concerning traits at different developmental stages of cotton genotypes.

**FIGURE 5:**
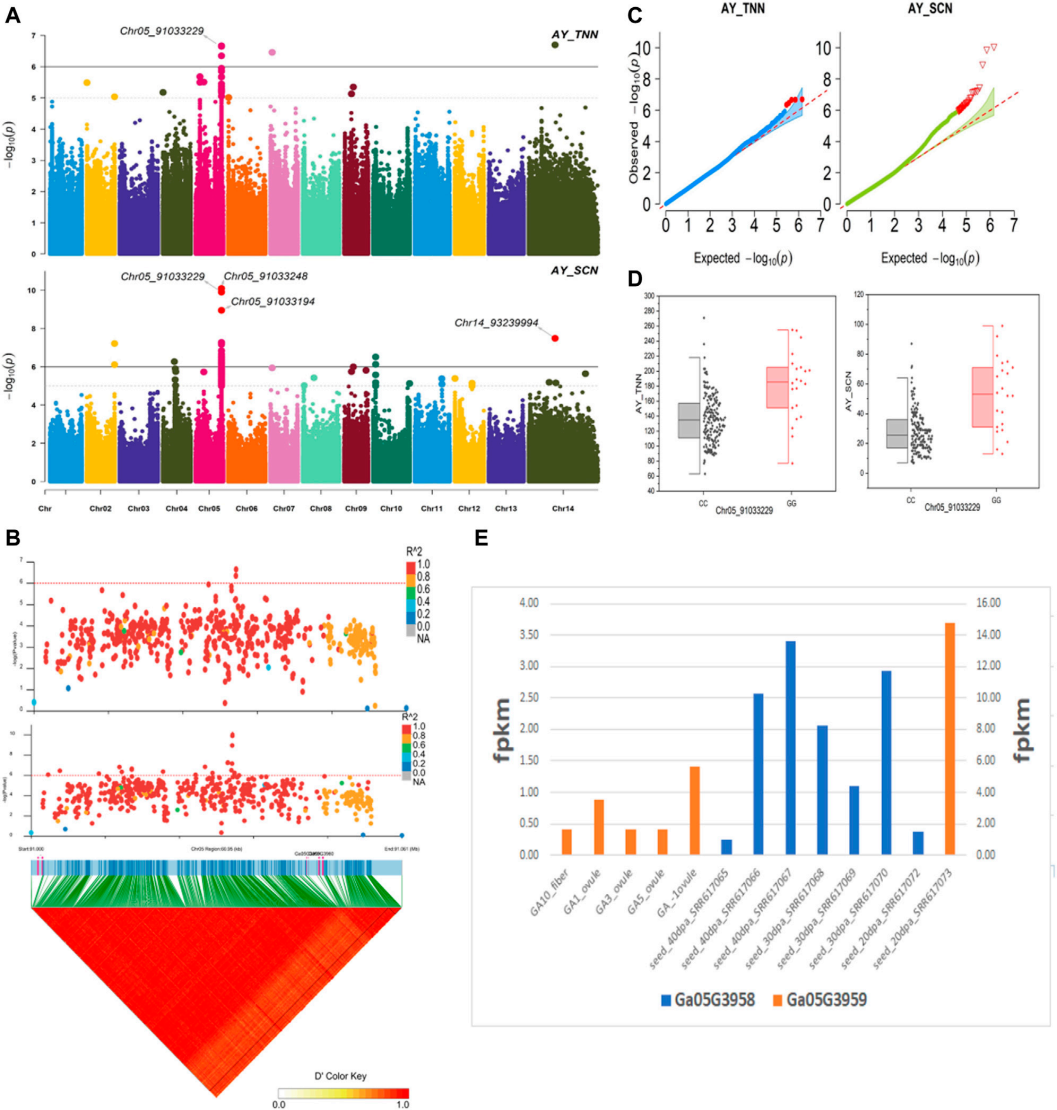
Genome-wide association studies for NEPS in *G. arboreum* **(A)** Manhattan plots for NEPs related traits: AY_TNN and AY_SCN; dotted-horizontal line in each plot depicts significance threshold value kept at [−log (*p*) > 6] **(B)** GWAS-LD block demonstrating haplotype region (60.95 kb) surrounding the displaying peak on chromosome Chr05 associated with NEPs related traits along with GWAS regional plot exposed key SNPs and nearby gene(s) in the region **(C)** Quantile-Quantile plots for NEPs related traits **(D)** Box plots revealing differences for NEPs related traits among two favorable haplotypes of SNP Chr05_91033229:AY_TNN (left) and Chr05_91033229:AY_SCN (right) **(E)** Expression levels of representative annotated genes *Ga05G3958* and *Ga05G3959* at different developmental stages of *G*. *arboreum*.

**FIGURE 6:**
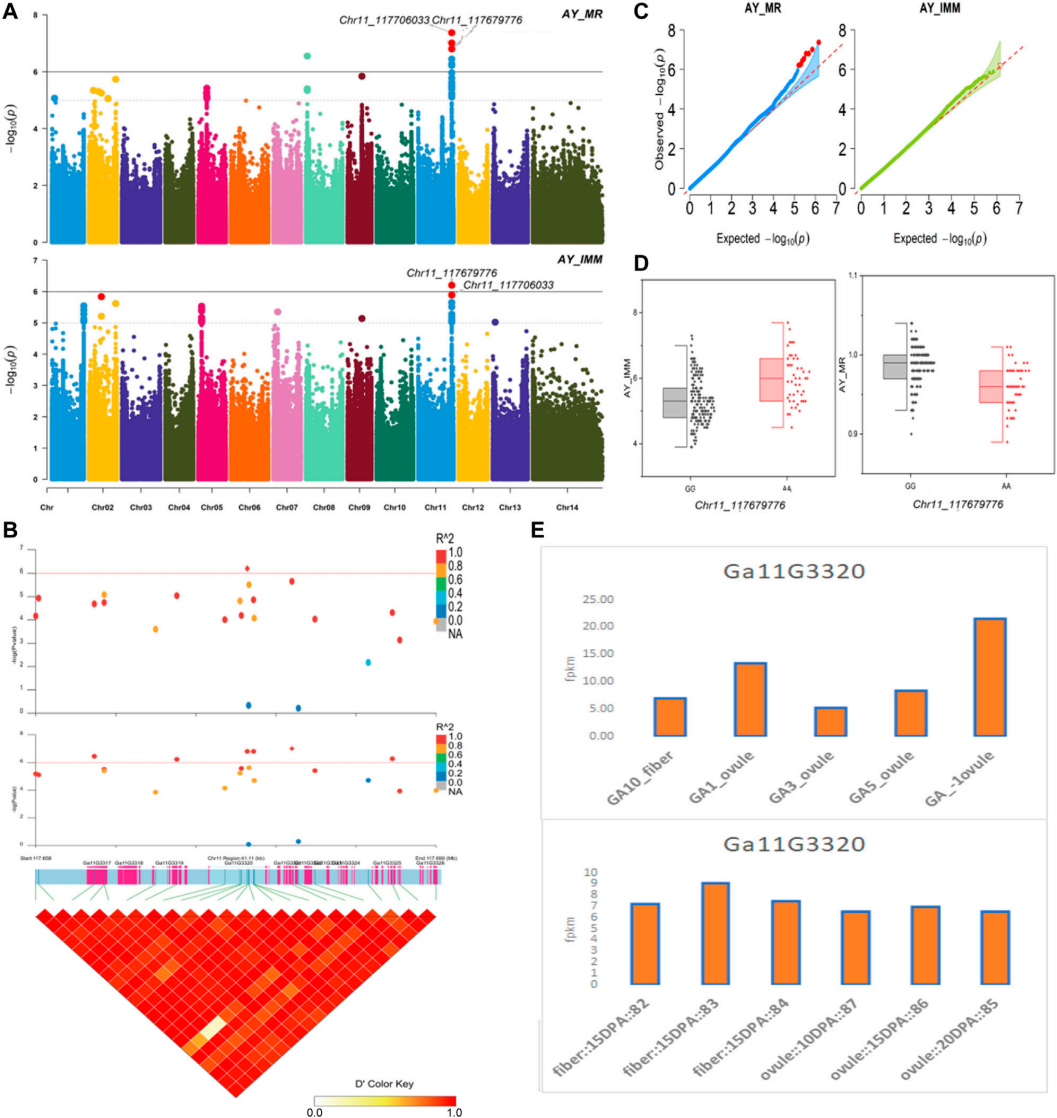
Genome-wide association studies for Maturity in *G. arboreum* **(A)** Manhattan plots for Maturity related traits: AY_MR and AY_IMM; dotted-horizontal line in each plot illustrates significance threshold value kept at [−log (*p*) > 6] **(B)** GWAS-LD block revealing haplotype region (41.11 kb) surrounding the exposed peak on chromosome Chr11 associated with Maturity related traits along with GWAS regional plot presenting key SNPs and nearby gene(s) in the region **(C)** Quantile-Quantile plots for Maturity related traits **(D)** Box plots demonstrating differences for Maturity related traits among two favorable haplotypes of SNP Chr11_117679776:AY_IMM (left) and Chr11_117679776:AY_MR (right) **(E)** Expression levels of representative annotated gene *Ga11G3320* at different developmental stages of *G*. *arboreum* from two different sources.

**FIGURE 7:**
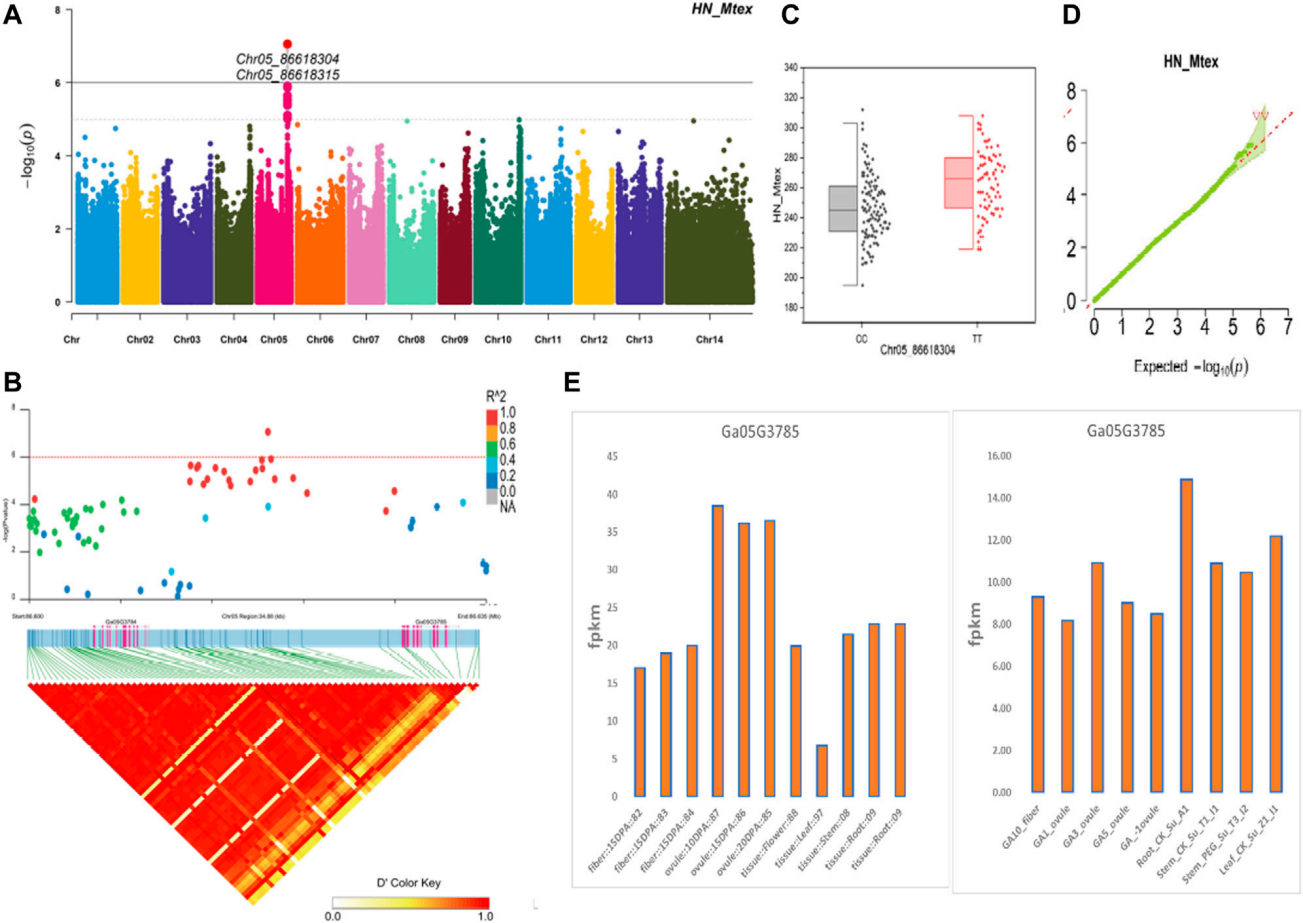
Genome-wide association studies for Fineness in *G. arboreum* **(A)** Manhattan plots for Fineness related trait HN_Mtex; dotted-horizontal line describes significance threshold value kept at [−log (*p*) > 6] **(B)** GWAS-LD block displaying haplotype region (34.88 kb) surrounding the displaying peak on chromosome Chr05 associated with Fineness related traits along with GWAS regional plot revealing key SNPs and nearby gene(s) in the region **(C)** Quantile-Quantile plots for Fineness related traits **(D)** Box plots presenting differences for Fineness related traits among two favorable haplotypes of SNP Chr05_86618304:HN_Mtex **(E)** Expression levels of representative annotated gene *Ga05G3785* at different developmental stages of *G*. *arboreum* from two different sources.

**FIGURE 8:**
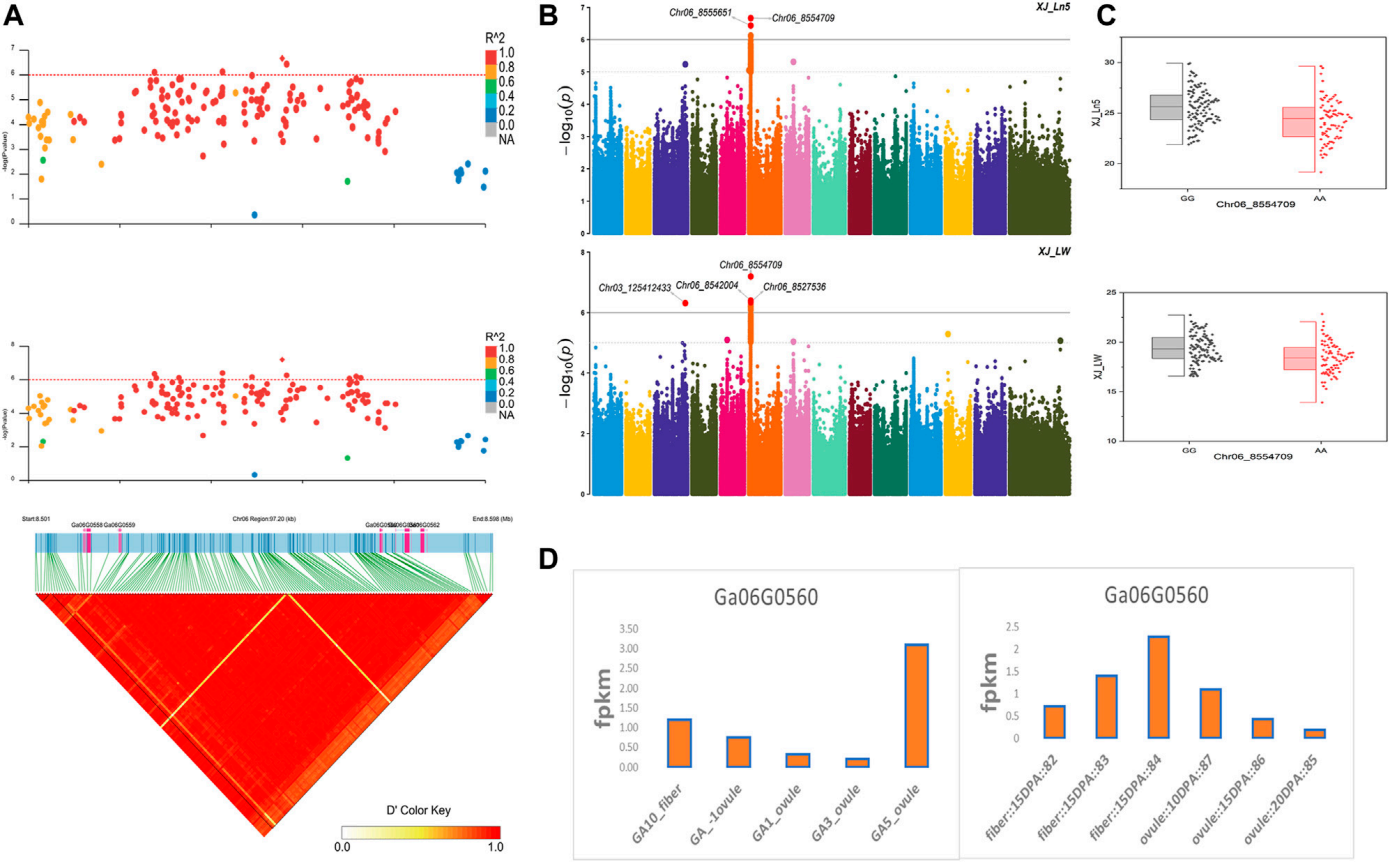
Genome-wide association studies for Length in *G. arboreum* **(A)** GWAS-LD block demonstrating haplotype region (97.20 kb) surrounding the exposed peak on chromosome Chr06 associated with Length related traits along with GWAS regional plot revealing key SNPs and nearby gene(s) in the region **(B)** Manhattan plots for Length related traits: XJ_Ln5 and XJ_LW dotted-horizontal line in each plot describes significance threshold value kept at [−log (*p*) > 6] **(C)** Box plots exhibiting differences for Length related traits among two favorable haplotypes of SNP Chr06_8554709: XJ_Ln5 and Chr06_8554709: XJ_LW **(D)** Expression levels of representative annotated gene *Ga06G0559* at different developmental stages of *G*. *arboreum* from two different sources.

The NEPs related two traits; AY_TNN and AY_SCN showed highly significant associations with pleiotropic lead SNP Chr05_91033229 at peak with log(*p*) > 6. The haplotypes of this SNP exhibited significant differences depicted *via* boxplots. The gene expression results gave following three genes namely, *Ga05G3958* and *Ga05G3959* in vicinity of this lead SNP. These candidate genes presented their higher expression levels regarding FPKM value in ovule, fiber, and seed tissues (Figure 5). The maturity related two traits; AY_MR and AY_IMM presented highly significant relationships with pleiotropic lead SNP Chr11_117679776 with log(*p*) > 6 found at peak of Manhattan plots. This lead SNP revealed distinct haplotypes mentioned in boxplots. The gene expression outcomes provided a gene viz, *Ga11G3320* in which this SNP was found. This candidate gene exhibited high FPKM values in almost all tissues of ovule, fiber, seed, stem, and root with highest expression in ovule at 20DPA (Figure 6). The fineness related trait Mtex gave maximum significant [log(*p*) > 6] association with the lead SNP i.e., Chr05_86618304. Its haplotypes showed significant differences from each other represented *via* boxplots. The gene expression findings provided a gene *Ga05G3785* in close vicinity of this lead SNP. This candidate gene presented high expression FPKM values which validated the selection of this gene as candidate one (Figure 7). The length related four traits namely, XJ_Ln5, XJ, XJ_LW, and XJ_UQLW displayed highly significant [log(*p*) > 6] associations with pleiotropic lead SNP Chr06_8554709 found at peak of Manhattan plots. The haplotypes boxplots of this lead SNP were significantly different. The relevant gene expression profiles showed a gene *Ga06G0559* in its close vicinity. This candidate gene presented its high expression FPKM values in ovule and seed tissues (Figure 8).

## Discussion

Cotton is a prominent natural source of fiber. The cultivated cotton species include both diploid and tetraploid genomes (Sarfraz et al., 2018). A general perception about present-day allotetraploid American Cotton (*Gossypium hirsutum)* confers to the diploid species, i.e., *Gossypium arboreum* and *Gossypium raimondii* (Cronn et al., 2002; Wendel and Cronn, 2003). Asian diploid cotton, a potential A-genome donor of upland cotton, is renowned for harboring many genetic factors coding high fiber quality-related features and resistances against several biotic and abiotic stresses (Shaheen et al., 2013). Due to the scarcity of available genetic divergence in the founder parents of global cotton cultivars, global climate change is posing continual threats to the development and survival of *G*. *hirsutum* cultivars. To restore the broad genetic base, it is a dire need to explore potential genetic diversity that might have eroded from the cultivated cotton collection during the breeding period to restore their broad genetic base.

The significance of fiber quality for premium textiles has prompted breeders to generate, and farmers to harvest new cotton varieties with superior fiber qualities. Several single fiber quality traits that HVI does not measure, i.e., fiber length distribution, short fiber content, maturity and fineness, have a considerable impact on processing performance. A critical concern in cotton research is the need for precise and accurate methods for measuring fiber quality traits. The dependency of maturity on fiber length, modeled using network analysis, confirmed their strong relationship. This relationship of length with fineness and maturity showed that the information for maturity and fineness of fiber is probably embedded in its length distribution (Paudel, 2012). It illustrates that the shorter fibers are immature than the longer fibers. Mature fibers have a secondary cell wall that is thicker in width, so less inclined to breakage during processing. Consequently, the breaking of immature fiber could increase short fibers for immature cotton.

The associations among the *G. arboreum* accessions were evaluated through hierarchical clustering based on genetic distance. There were three distinct clusters. The distribution of accessions in the distant Clusters 2 and 3 reflect their sizeable geographic distance, i.e., Southwest China and Northern China. Additionally, the closeness between Cluster 1 and Cluster 3 confirms that *G*. *arboreum* originated and expanded into the Yangtze River region from South China. A general inference drawn from this finding was that *G*. *arboreum* species first originated in South China and then extended to the Yellow and Yangtze River regions (Guo et al., 2006). The three clusters, with Clusters 1 and 2 being close together and Cluster 3 being farther away, were also revealed by PCA. This clustering corroborates earlier findings (Yinhua et al., 2018).

The efficacy of GWAS decreases when the variations in the population under study increase (Tyagi et al., 2014). Also, the power of structure-based studies to detect single gene effects decreases with increases in population differences (Flint-Garcia et al., 2005). *G*. *arboreum* is better than tetraploid cotton for identifying trait-related genes *via* GWAS due to its low population differentiation and smaller genome. Hence, *G*. *arboreum* is useful for exploring population differences and genetic diversity to facilitate identifying genes significantly associated with critical traits (Yinhua et al., 2018).

LD analysis plays a unique role in GWAS in evaluating the density and number of associated markers between loci where LD persists. Factors affecting LD include population size, genetic diversity, admixture level, marker system, mating design, and selection techniques (Flint-Garcia et al., 2003; Stich et al., 2006). The LD decay distance in cross-pollinated crops such as maize (1–100 kb) is lower than in self-pollinated species such as cotton (Flint-Garcia et al., 2003), and also *Arabidopsis thaliana* (∼250 kb) (Nordborg et al., 2002), rice (75–500 kb) (Mather et al., 2007), and soybean (100–600 kb) (Hyten et al., 2007). However, LD decay was 25 cM in tetraploid cotton based on microsatellites (Abdurakhmonov et al., 2009). We determined an LD decay distance of ∼115.5 kb with *r* ^2^ = 0.42, similar to other reports on self-pollinated species. In the last two decades, GWAS has been extensively used to map various quantitative traits in plants, being considered an important milestone (Zhao et al., 2014). The power of GWAS depends on rich genetic diversity, accurate phenotypic data, marker density, and adequate statistical methods.

The gene *Ga05G3958* found in close vicinity of lead SNP Chr05_91033229 associated with NEPs related traits, encodes a protein S-norcoclaurine synthase 2 (Q4QTJ1) plays role in defense mechanism again pathogens reported earlier in *Opium poppy* (Lee and Facchini, 2010). The expression of this protein was found in its roots, leaves, stem, flower buds and germinating seeds (Samanani and Facchini, 2001) like our findings having this gene with higher expression in seed tissues at different stages for seed coat nep (SCN) and total nep count (TNN). The other NEPs related gene *Ga05G3959* encodes Nucleoporin nup107 protein reported in Fission yeast. As the name indicates, the protein is located in nucleus and related functions involved transport of mRNA, rRNA, and various proteins across nuclear envelope (Gaudet et al., 2011). In our findings, this gene gave its higher expression in seed tissues at various stages. The gene *Ga05G3785* was found in vicinity of lead SNPs related to fineness gave a higher expression in all tissues of fiber, ovule, and seed at different stages. It was previously described in *Arabidopsis* encoding for DJ-1 homolog B (DJ1B) protein which is involved in the oxidative stress response (Kwon et al., 2013). Two genes *Ga03G2389* and *Ga03G2390* were observed in close vicinity of lead SNPs related to length and NEPs, respectively*.* They were previously described in *Arabidopsis*. *Ga03G2389* encodes LIM domain-containing protein (WLIM1) which binds to actin filaments to promote cross-links to form thick bundles (Papuga et al., 2010), strengthening the fibers and ultimately may play roles in its lengthening. *Ga03G2390* encodes ADP-ribosylation factor-like A1C protein from GTPase family (Nishikiori et al., 2011; Du et al., 2018), involves in GTP binding is located in nucleus, plasma membrane, male gametophyte (pollen tube) during maturation, and fiber during development stages. A length related gene *Ga06G0559* was found in vicinity of Chr06_8554709 lead SNP encodes Sporulation-specific glucan 1,3-beta-glucosidase (SPR1) protein found in Baker’s yeast. This enzyme is expressed in later stages of sporulation for the modification of glucan linkages in order to strengthen the ascospore wall or providing it plasticity (Gaudet et al., 2011). The reported function of cell wall organization in yeast spores is validating this gene association with our length related traits for provision of shape, strength, and plasticity to fiber.

*Ga02G1729*, *Ga02G1738*, *Ga02G1741*, *Ga08G0324*, *Ga11G3317*, *Ga11G3319, Ga11G3320*, and *Ga11G3321* were found nearby the lead SNPs related to NEPs and maturity. *Ga08G0324*, *Ga02G1738*, *Ga11G3317*, *Ga11G3319*, and *Ga11G3320* were previously described in *Arabidopsis*. *Ga02G1729* encodes RETICULATA protein and is located in chloroplastic DNA. It may play a prominent role in leaf development as it is key for mesophyll cell division during initial leaf organogenesis. It is highly expressed during embryo development and in leaf primordia, margins of fully expanded leaves, stipules, lamina, root tips, and stamens (Barth and Conklin, 2003; González-Bayon et al., 2006; Pérez-Pérez et al., 2013). *Ga08G0324* encodes DNA damage and repair/toleration protein (DRT100). *Ga02G1738* encodes mediator of RNA polymerase II transcription subunit protein. It regulates flowering time and plant defense and is involved in pollen tube growth (Lalanne et al., 2004; Kidd et al., 2009). *Ga11G3317* (non-synonymous mutation) encodes FAR1-related sequence 5 (FRS5) protein. It is involved in zinc ion binding and transcription regulation and is expressed in hypocotyl tissues, leaves, stems, and flowers. It is upregulated in hypocotyl tissues (Lin and Wang, 2004). *Ga11G3320* encodes membrane-anchored ubiquitin-fold protein 1 (MUB1). It is involved in stability at temperatures >90°C and is located at the plasma membrane (Downes et al., 2006). *Ga11G3319* (non-synonymous mutation) encodes galacturonosyltransferase 13 (GAUT13). It is involved in pectin biosynthesis in cell walls, pollen tube growth, and pollen development, and it is expressed in flowers, roots, stems, and leaves (Caffall et al., 2009).

GO annotation revealed that the abovementioned genes were associated with several biological processes, cellular components, and molecular functions. These genes encode for proteins involved in the transport/metabolism of amino acids, coenzymes, inorganic ions, lipids and carbohydrates, cell wall/membrane/envelope/ribosomal structure and biogenesis (deposition), energy production or conversion, intercellular trafficking, secretion, vesicular transport, transcription, translation, post-translational modification, protein turnover, chaperons, replication, recombination, repair, signal transduction mechanisms, and general functions prediction.

## Conclusion

Continuous improvements of cotton fiber quality are required to maintain the superiority of cotton fiber over manmade yarn. Significant research is needed to improve the measurement accuracy of key cotton fiber traits. Basically, 4 categories of 17 AFIS-related fiber quality traits, including NEPS, Fiber Length, Fiber Fineness and Fiber maturity, were evaluated in the current study. Rapid and precise measurement of quality traits will help cotton breeders quickly select key traits to develop varieties with superior fiber quality suitable for industrial use. A substantial amount of highly significant SNP markers (QTNs) for these traits were identified and further validated *via* gene expression analysis. Highly significant genes present in the vicinity of these key SNPs were considered as candidate genes. These compendia connecting traits, genes and cell types may allow further prioritization of genes in GWAS loci to enable mechanistic studies. These identified QTNs can possibly be helpful to cotton breeders regarding fiber quality improvement as well as revival of eroded genetic factors of *G*. *hirsutum via* introgression and marker-assisted breeding approaches.

## Data Availability

All raw sequencing available at the NCBI BioProject database under accession number PRJNA349094.

## Supplementary Material

Supplementary Figure S1 Summary of correlations among different fiber quality traits of *G*. *arboreum* represented in upper triangle. The density distribution of all accessions for different traits evaluated in different environments is displayed at diagonal with distinct colors (Pink: Anyang, Green: Henan, Blue: XJ: Xinjiang). In the lower triangle, bivariate scatterplots of all investigated traits are presented. Boxplots are illustrating the presence of variability among all experimented accessions. Central box displays middle half data extending from upper to lower quartile whereas, a horizontal line is drawn to show the median. The endpoints of vertical projections designate minimum and maximum data points until the presence of outliers. The rows at the bottom depict frequency distributions of each environment for all the traits.

Supplementary Figure S2 Quantitative Trait Nucleotides (QTNs) rich regions with significant (*p* < 0.00001) associations among fiber quality traits across all the chromosomes of Asiatic Cotton.

Supplementary Figure S3 Heat map of gene expression (fpkm) levels of 113 genes in the vicinity of lead SNPs based on transcriptome data from two sources.
