# Supplementary figures and images for: Genetic Factors Underlying Single Fiber Quality in A-Genome Donor Asian Cotton (Gossypium arboreum)
Source: Front Genet. 2021 Dec 7;12:758665. doi: 10.3389/fgene.2021.758665 (PMC8689003; doi:10.3389/fgene.2021.758665)

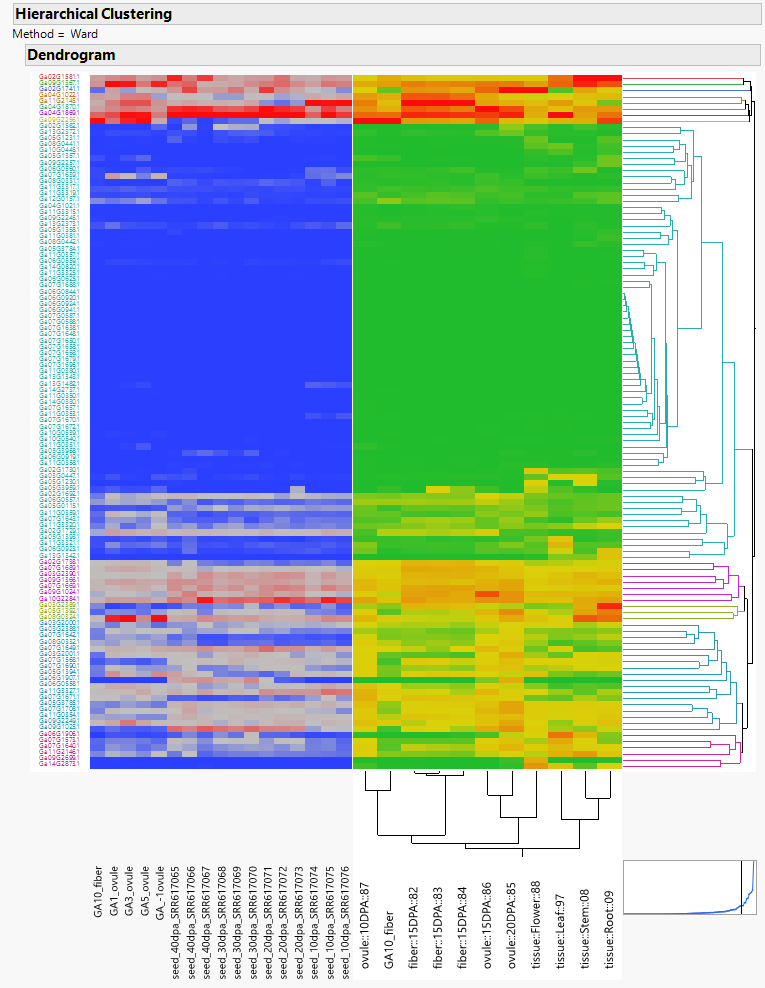

Supplement: Supplementary file 1 [file Image3.TIFF]

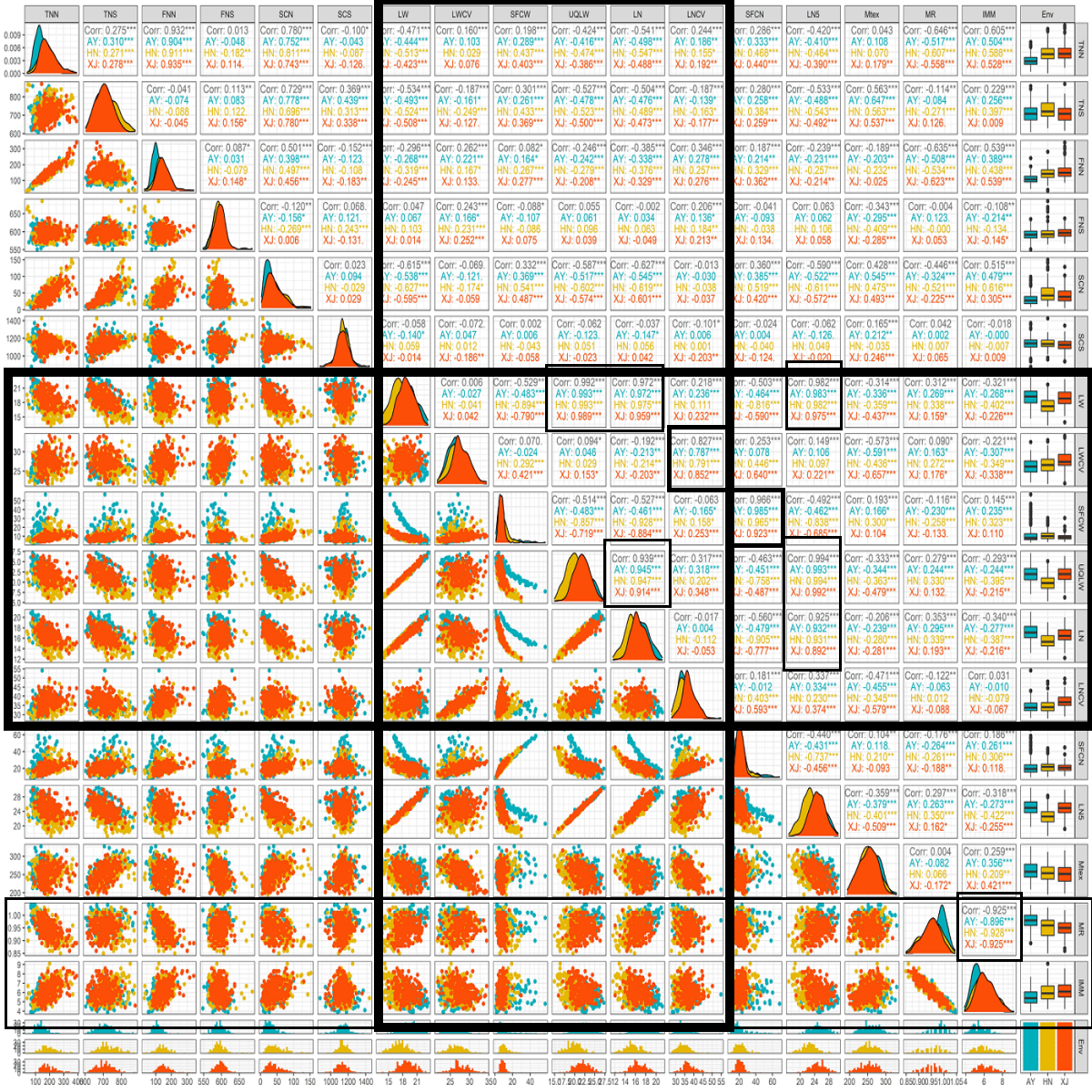

Supplement: Supplementary file 2 [file Image1.TIFF]

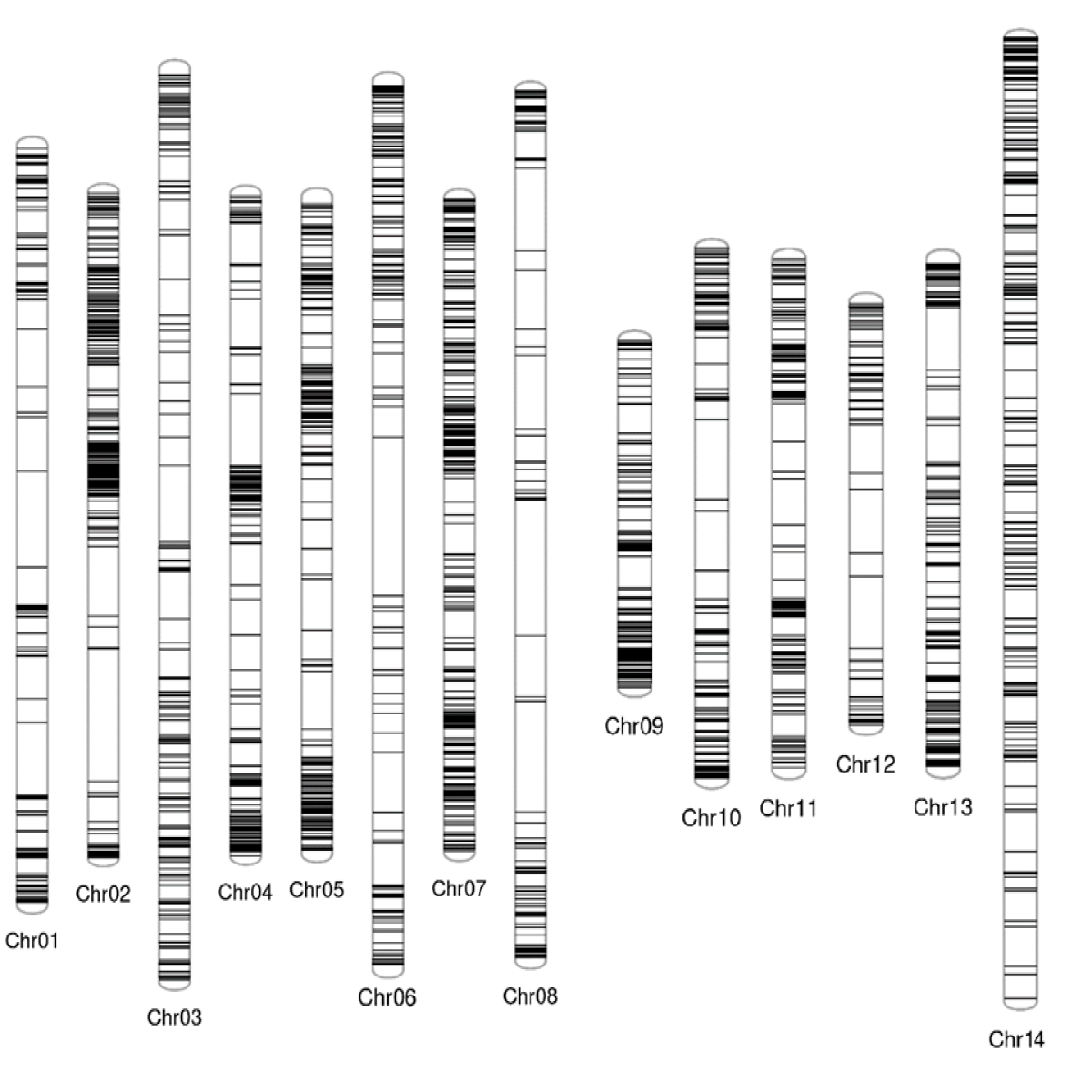

Supplement: Supplementary file 3 [file Image2.TIF]
